# Revolutionizing stress-related disorder regulation through neuroinformatics and data analysis: An editorial

**DOI:** 10.3934/Neuroscience.2023019

**Published:** 2023-09-04

**Authors:** Arosh S. Perera Molligoda Arachchige, Yash Verma

**Affiliations:** Department of Biomedical Sciences, Humanitas University, Milan, Italy

In recent years, stress-related disorders have become a pressing global health concern, affecting millions of individuals worldwide. The profound impact of these disorders on mental well-being and overall quality of life has propelled the scientific community to explore innovative approaches to better understand, diagnose and treat them. In this endeavor, neuroinformatics and data analysis have emerged as powerful tools that hold immense promise in revolutionizing our understanding and regulation of stress-related disorders. Inspired by this special issue of *AIMS Neuroscience*, we would like to provide a thoughtful perspective on the role of neuroinformatics and data analysis in the regulation of stress-related disorders [1].

Neuroinformatics, a multidisciplinary field that combines neuroscience, computer science and informatics, has the potential to unlock the secrets of the human brain. By integrating vast amounts of neuroimaging data, genetic information and clinical records, neuroinformatics allows researchers to unravel the complex neurobiological mechanisms underlying stress-related disorders. Neuroimaging techniques such as functional magnetic resonance imaging and positron emission tomography enable researchers to visualize and quantify brain activity and structural changes associated with stress [2]. By utilizing neuroinformatics tools, scientists can analyze these datasets in unprecedented ways, identifying patterns and biomarkers that can aid in the early detection and diagnosis of stress-related disorders.

In addition, the availability of vast amounts of data in the digital age has necessitated the development of advanced data analysis techniques. By employing machine learning algorithms and statistical models, researchers can sift through massive data sets to identify meaningful correlations, predict outcomes and develop personalized treatment approaches [3]. Data-driven approaches have the potential to revolutionize the regulation of stress-related disorders. By aggregating and analyzing data from diverse sources, including electronic health records, wearable devices and mobile applications, researchers can gain a comprehensive understanding of an individual's stress response and its impact on mental health [4],[5]. This wealth of information can inform the development of targeted interventions and preventive strategies tailored to the unique needs of each patient.

One of the critical challenges in managing stress-related disorders is the timely identification of individuals at risk or those already experiencing symptoms. Neuroinformatics, coupled with data analysis, offers the potential for earlier detection and diagnosis of stress-related disorders, improving outcomes and reducing the burden on healthcare systems. Integration of multi-omics data in stress-related disorders presents both challenges and opportunities. It allows for a comprehensive analysis of various biological layers, such as genomics, epigenomics, transcriptomics and proteomics, providing a deeper understanding of the molecular mechanisms underlying stress-related disorders. However, the complexity and heterogeneity of multi-omics data pose challenges in data integration, interpretation and validation, requiring advanced computational and analytical approaches to unlock the full potential of this valuable resource. Through the integration of diverse data sources, including neuroimaging, genomic, proteomic and behavioral data, researchers can identify reliable biomarkers associated with stress-related disorders [12]. These biomarkers can serve as early indicators, allowing for proactive intervention and preventive measures to mitigate the progression of these disorders [6],[7]. Furthermore, each individual responds uniquely to stress, necessitating personalized treatment approaches. Neuroinformatics and data analysis have the potential to uncover the underlying mechanisms of stress-related disorders and identify factors that contribute to their development and progression. By understanding these individual variations, clinicians can tailor treatments to maximize effectiveness and minimize adverse effects [8]. Machine learning algorithms can aid in predicting treatment response based on an individual's neurobiological profile, genetic markers and other relevant factors [9]. This precision medicine approach holds promise in guiding treatment decisions, optimizing medication selection and improving therapeutic outcomes.

While neuroinformatics and data analysis offer great promise, they also present ethical considerations and challenges. Safeguarding patient privacy, ensuring data security and addressing potential biases in data collection and analysis are crucial aspects that require careful attention [10],[11]. Furthermore, the accessibility and affordability of neuroinformatics tools and data analysis techniques need to be addressed to ensure equitable access for all communities. Collaboration between researchers, policymakers and healthcare providers is essential to develop guidelines and regulations that promote responsible and ethical use of these technologies.

In conclusion, neuroinformatics and data analysis are revolutionizing our understanding and regulation of stress-related disorders. We believe this special issue of *AIMS Neuroscience* is one of the perfect venues to spark evidence-based discussion on this topic. By harnessing the power of advanced computational tools and the integration of diverse data sources, we can unlock the mysteries of the human brain and develop personalized approaches to prevention, diagnosis and treatment. However, it is imperative that we address ethical considerations and ensure equitable access to these technologies, ensuring that they are used responsibly and for the benefit of all individuals affected by stress-related disorders. By embracing the potential of neuroinformatics and data analysis, we can pave the way for a future where stress-related disorders are effectively regulated, improving the lives of countless individuals around the world.
